# Short-term aspirin and statin chemoprophylaxis did not reduce the risk of developing advanced adenomatous polyps in Black patients

**DOI:** 10.1186/s12876-021-01966-4

**Published:** 2021-10-17

**Authors:** Benjamin D. Renelus, Devika Dixit, Phuong T. Nguyen, Kingsley K. Njoku, Parth B. Patel, Katiria Pintor-Jimenez, Fengxia Yan, Jonathan M. Buscaglia, Kevin E. Woods, Daniel S. Jamorabo

**Affiliations:** 1 Division of Gastroenterology and Hepatology, Johns Hopkins University School of Medicine, Baltimore, MD USA; 2 Department of Internal Medicine, University of Florida, Gainesville, FL USA; 3 Department of Internal Medicine, Morehouse School of Medicine, Atlanta, GA USA; 4 Department of Community Health and Preventative Medicine, Morehouse School of Medicine, Atlanta, GA USA; 5 Division of Gastroenterology and Hepatology, Stony Brook Medicine, 101 Nicolls Road, Stony Brook, NY HSC T17-06011794 USA; 6 Therapuetic GI Associates, LLC, Atlanta, GA USA

**Keywords:** Chemoprevention, Advanced adenomatous polyps, Black patients

## Abstract

**Background:**

Chemoprevention of colorectal neoplasia with aspirin and statins is under-investigated in Black patients. Since Black patients suffer disproportionately from colon cancer incidence and mortality compared to other populations, we investigated the utility of aspirin and statin in reducing advanced adenomatous polyp (AAP) risk in Black patients.

**Methods:**

We carried out a retrospective cohort study of screening colonoscopies performed at a large urban academic center from 1/1/2011 through 12/31/2019. We analyzed self-identified Black patients with > 1 colonoscopy and no personal history of either inflammatory bowel disease or colon cancer syndromes. Our primary endpoint was first AAP development after index colonoscopy among Black patients taking both aspirin and a statin compared to those taking one or neither medication. We used multivariate logistic regression modeling to investigate our outcomes.

**Results:**

We found data on chemoprophylaxis use in 560 patients. The mean observation period between index colonoscopy and AAP identification was 4 years. AAP developed in 106/560 (19%) of our cohort. We found no difference in AAP risk among Black patients taking both chemoprevention medications compared to partial or no chemoprophylaxis (20% vs 18% respectively, p = 0.49). This finding remained after adjusting for age, body mass index, and tobacco use (odds ratio 1.04, 95% CI 0.65–1.67; p = 0.87).

**Conclusions:**

Short-term aspirin-statin chemoprevention did not reduce the risk of AAP development in our cohort of Black patients. Larger and long-term prospective investigations are needed to investigate the utility of chemoprophylaxis in this population.

*Trial Registration*: Not applicable.

## Introduction

Most colorectal cancers (CRCs) are thought to develop from pre-malignant adenomatous polyps [1, 2] with advanced adenomatous polyps (AAP) carrying the highest risk for malignant transformation. Despite an overall decrease in new CRC cases in recent years, some studies show persistent racial disparities in the incidence and mortality thereof, even among those with early-onset CRC [3, 4]. Black patients are more likely than White patients to develop AAP and to be diagnosed with stage IV CRC [5, 6]. In response, a United States multi-society task force advised initiating colon cancer screening at a younger age for Black patients that was later reinforced by the American Cancer Society [7].

Chemoprevention, also known as chemoprophylaxis, aims to reduce CRC incidence by exploiting the effects of different medications on the cell cycle. For example, aspirin can theoretically slow tumor growth by reducing downstream inflammation while statins can prevent tumor spread by arresting the cell cycle [8]. Publications on aspirin-statin chemoprophylaxis have been inconclusive due to lack of population diversity and generalizability [9, 10]. In addition, the role of chemoprevention in reducing the development of high-risk AAP has not been well studied. Though animal experiments have shown a synergistic effect between non-steroidal anti-inflammatory drugs (NSAIDS) and statins for chemoprevention of colorectal tumors [11, 12], research into AAP chemoprevention in Blacks is lacking. Our goal was to investigate the utility of aspirin and statins in reducing the risk for developing AAP among Black patients.

## Methods

### Participants and setting

We performed a retrospective review on screening colonoscopies done at a large urban teaching hospital from 1/1/2011 through 12/31/2019. We included all adults aged 50 years and above who had undergone multiple colonoscopies due to average or above average risk for colorectal cancer. We excluded patients with only one colonoscopy on record, a personal history of hereditary polyposis or non-polyposis colon cancer syndromes, a personal history of CRC, CRC found on index colonoscopy, and personal history of inflammatory bowel disease. We also excluded patients that had documented inadequate or poor bowel prep on endoscopy reports. We defined poor or inadequate bowel prep as Boston Bowel Prep Score < 6 or specific colon section score < 2 if prep quality not explicitly written within the endoscopy report. Our study was approved by our institutional review board (#1638337-1).

### Procedures and endpoints

Data was obtained from the electronic medical record (EMR) and comprised of demographic and clinical information. Physician researchers uniformly collected patients’ age, sex, self-reported race, medication use, colonoscopy reports, and pathology reports detailing polyp size and histopathology. We defined AAP as a polyp > 9 mm in size or any size polyp with either villous changes or high-grade dysplasia (HGD) confirmed on histology. Our primary endpoint was AAP risk after index colonoscopy among Black patients taking both aspirin and statins, compared to those taking neither or only one of the two medications. Incident metachronous AAP was documented at the initial post-index colonoscopy. Secondary endpoints included comparative AAP risk after index colonoscopy among Black patients with or without AAP or sessile serrated polyp (SSP) on index colonoscopy and those with 0–2 neoplastic polyps and > 2 polyps on index colonoscopy. We considered the first documented colonoscopy to be the index colonoscopy while the surveillance interval to the finding of a lesion was the first colonoscopy done after the index one. Statin use was confined to high intensity statins, specifically rosuvastatin and atorvastatin. Medication use was confirmed through documentation with linked pharmacy data. The medication administrative record was reviewed at the time of index throughout the observation period at post index colonoscopy, thereby allowing us to calculate how long patients were prescribed aspirin and statins between procedures. We assumed that patients were compliant with their prescriptions for the intervals between their index and subsequent colonoscopies.

### Sample size and statistical analysis

We assumed the prevalence of AAP among Blacks above the age of 50 would be 9% based upon findings reported from Friedenberg’s group [13]. We also assumed that combined aspirin and statin use would reduce the risk of AAP by 6% based upon the impact of continuous statin use on AAP development observed by Siddiqui’s group [14]. Using an alpha of 0.05 and beta of 0.2, a total of 490 participants, 245 in each group would be needed to detect a difference between the two groups with a power of 0.8.

Descriptive statistics were used to describe the characteristics of the patients. Means with standard deviation were used for numerical variables and frequency with percentage were used for categorical variables. Chi-square or Fisher’s exact test were used to compare the percentages among categorical variables while two sample t-testing was used to compare the means and medians of the numerical variables. Univariate logistic regression model was used to construct Odds Ratio (OR) plots. Multivariate logistic regression modeling accounting for age, body mass index (BMI), and tobacco use was performed to further investigate our findings. The SAS 9.4 software (SAS Institute Inc., Cary, NC, USA) was used for all the data analysis and p < 0.05 was considered to be statistically significant.

## Results

### Baseline characteristics

A total of 826 Black patients were identified after initial chart review. Documentation regarding chemoprevention was present in 560 (67.8%) of our cohort. A total of 295 (53%) patients were documented to be taking both aspirin and a statin while the remaining 265 (47%) were taking either one or neither medication. AAP arose in 106 (19%) of the targeted cohort. The mean age at first colonoscopy was 59 years. A summary of the baseline characteristics can be found in Table 1.

**Table 1:** Baseline demographics

Advanced adenomatous polyp on subsequent colonoscopy?				
Variables		No (number/percent)	Yes (number/percent)	p-value
Sex	Female	411 (55.09)	108 (47.79)	0.05
	Male	335 (44.91)	118 (52.21)	
Statin use	No	221 (42.02)	41 (33.88)	0.10
	Yes	305 (57.98)	80 (66.12)	
Tobacco use	Never	250 (33.88)	58 (25.89)	< 0.001
	Quit	249 (33.74)	109 (48.66)	
	Yes	239 (32.38)	57 (25.45)	
Aspirin use	No	252 (33.78)	79 (34.96)	0.74
	Yes	494 (66.22)	147 (65.04)	
Documented race	Black/African-American	641 (85.92)	185 (81.86)	0.13
	Non-Black	105 (14.08)	41 (18.14)	
Statin and aspirin use	Both	236 (51.98)	59 (55.66)	0.56
	Part/None	218 (48.02)	47 (44.34)	
Age at first colonoscopy		59.04 (8.99)	59.54 (8.05)	0.43
Body mass index		30.21 (7.17)	31.26 (7.49)	0.07

### Endpoint

**Fig. 1:**
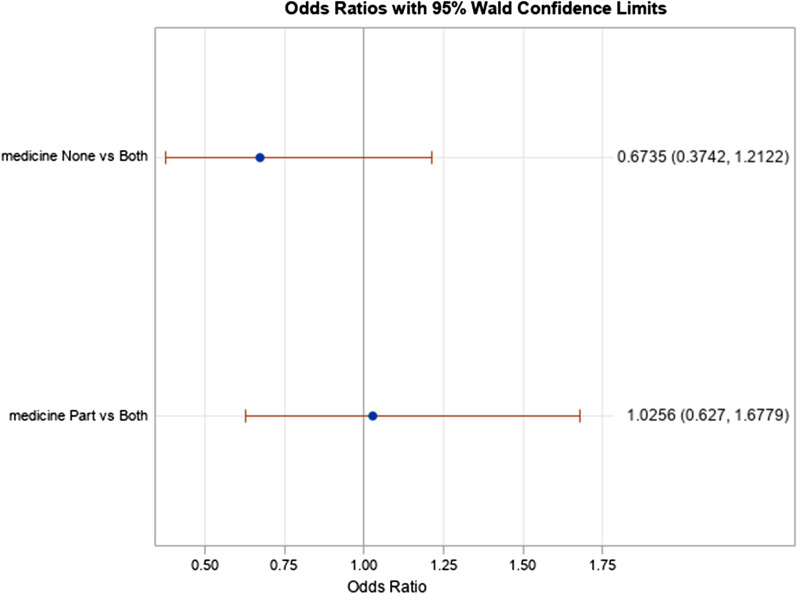
Risk of advanced adenomatous polyp in black patients on dual, partial, or no chemoprevention on subsequent colonoscopy

**Fig. 2:**
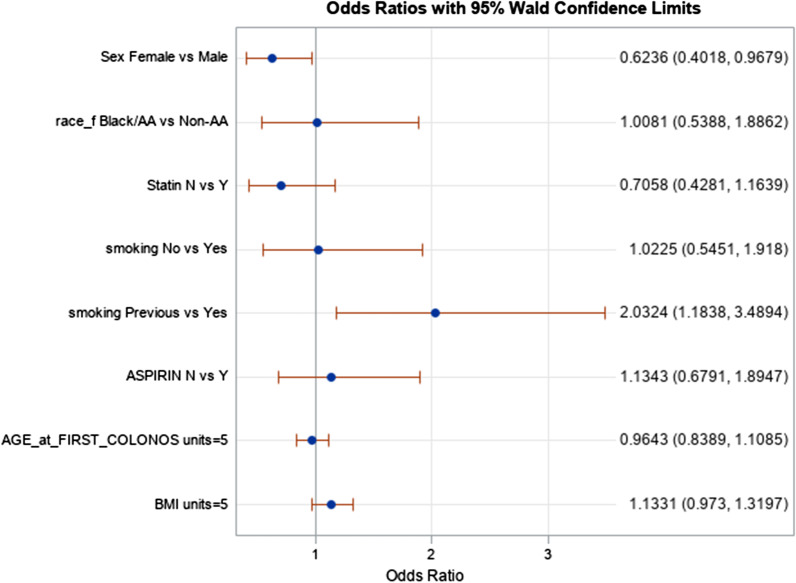
Risk of advanced adenomatous polyp development

The mean observation period between index colonoscopy and identification of AAP was 3.77 ± 2.91 years, which approximated how long patients were on chemoprevention or not. Women were less likely than men to have AAP on post-index colonoscopy (OR 0.62; 95% CI 0.40—0.97; p = 0.02), while former smokers were more likely to develop AAP compared to non-smokers or active smokers. We found no difference in AAP risk among Black patients taking both chemoprevention medications compared to partial or no chemoprophylaxis (20% vs 18% respectively, p = 0.49). This finding remained after adjusting for age, BMI, and tobacco use (OR 1.04; 95% CI 0.65–1.67; p = 0.87). AAP on index colonoscopy significantly increased the odds of developing AAP in subsequent colonoscopy even after adjusting for number of neoplastic polyp and presence of SSP on index colonoscopy (OR 4.08; 95% CI 2.75–6.06; p < 0.001). There was a trend toward a significant increase in AAP development on univariate analysis when > 2 adenomatous polyps were identified on index colonoscopy, but this trend was weakened with multivariate analysis. AAP risks are summarized in Tables 2, 3 and in Figs. 1, 2.

**Table 2:** Risk factors for advanced adenomatous polyps (AAP)

Variable	Number of patients	AAP on subsequent colonoscopy		Univariate analysis		Multivariate analysis	
		No (n = 641)	Yes (n = 185)	T-test or Chi-squared	p-value	Odds ratio (95%CI)	p-value
Mean Age at First Colonoscopy	826	59.5 (SD 8.92)	59.8 (SD 7.9)	0.47	0.64	0.99 (0.96–1.02)	0.49
Body Mass Index	754	30.3 (SD 7.1)	31.1 (SD 7.5)	1.25	0.21	1.02 (0.99–1.05)	0.24
Never Smoker	249	204 (32.2%)	45 (24.5%)	–	–	1.04 (0.52–2.1)	0.91
Former Smoker	309	216 (34.1%)	93 (50.5%)	–	–	2.4 (1.4–4.4)	0.003
Current Smoker	260	214 (33.7%)	46 (25.0%)	–	–	Reference	–
Concomitant Aspirin and Statin	295	236 (52.0%)	59 (55.7%)	0.47	0.50	1.0 (0.65–1.7)	0.87
Aspirin or Statin or Neither	265	218 (48.0%)	47 (44.3%)	–	–	Reference	–

**Table 3:** Risk of AAP Based Upon Index Findings

	Eventual Advanced Adenoma		Univariate Analysis		Multivariate Analysis	
	No (N = 746)	Yes (N = 226)	OR (95% CI)	p-value	OR (95% CI)	p-value
Any adenoma on index colonoscopy						
0–2	348 (62.82)	88 (54.66)	Reference		Reference	
> 2	206 (37.18)	73 (45.34)	1.40 (0.98–2.00)	0.0624	1.02 (0.69–1.51)	0.9126
Sessile serrated adenoma on index colonoscopy						
Yes	18 (3.23)	159 (96.36)	1.13 (0.44–2.90)	0.799	0.74 (0.28–1.97)	0.5514
No	539 (96.77)	6 (3.64)	Reference		Reference	
Advanced adenoma on index colonoscopy						
Yes	190 (32.93)	108 (63.91)	3.61 (2.52–5.16)	< 0.001	4.08 (2.75–6.06)	< 0.001
No	387 (67.07)	61 (36.09)	Reference		Reference

## Discussion

To our knowledge, this is one of the largest studies on this topic involving a Black cohort as primary data. We found no reduction in AAP risk for Black patients on short-term combined aspirin-statin chemoprevention on interval post-index colonoscopy. We also found that Blacks with AAP on index colonoscopy were significantly more likely to develop a metachronous recurrence. Multiple genes have been identified in the transition of normal colonic mucosa to adenomatous polyps including the adenomatous polyposis coli (APC) and multiple intestinal neoplasia genes. These changes affect cell proliferation and DNA restoration, which leads to increased cell turnover at the level of the adenomatous crypt.

Aspirin and statins affect multiple targets in the colorectal tumor pathogenesis pathway. Aspirin inhibits aberrant *Wnt* pathway activation by APC in addition to reducing inflammatory cytokine release and prostaglandin-E2 induced stem-cell programming [15]. Animal models have shown statins to be effective at reducing colon carcinogenesis, which is related to statins’ inhibition of hydroxy-3-methylglutaryl coenzyme A reductase that is up-regulated in tumors [11]. Still, the role of aspirin and statins in chemoprevention of CRC remains inconclusive and their ability to affect other carcinogenesis pathways has yet to be elucidated [16].

Our findings are consistent with Park, et al. who found a significant reduction in CRC incidence among Japanese and White men with history of aspirin use, but this observed association was not apparent among their Black patients [17]. Other authors, however, have reported findings at odds with our own. For example, Ruder, et al. used the National Institute of Health-AARP (NIH-AARP) diet and health study participants—including over 10,000 Black participants—for their observational study and found a significant reduction in CRC incidence among daily and weekly aspirin users compared to non-users [18]. We commend the comprehensive analysis the authors undertook to provide this important finding, but we also acknowledge key differences between our studies. The investigators did not perform a subset analysis for Black patients and they used questionnaires to determine medication used, whereas we studied Black patients exclusively and used pharmacy-linked data confirming medication use to avoid potential recall bias. Furthermore, our study endpoint was AAP development while theirs was CRC development. Siddiqui, et al. found in their study of 400 Black patients that statins reduced the incidence of AAP after index colonoscopy during an observation period of up to five years [14]. Unlike our study, however, their cohort was composed of 85% men while ours was 47% men; this is noteworthy since prior studies have documented lack of efficacy in CRC chemoprevention among women [17].

There are notable limitations to our study. Our single-center retrospective design lends itself to selection bias and challenges of population generalizability. This may be reflective in our relatively high rate of AAP development. Persistent confounders such as differences in CRC risk based on family history, health literacy, outside medical care, sex, and tobacco use remain a concern. Although the medical records allowed linkage to pharmacy prescription data, use and compliance could not be confirmed. Thus, we could calculate how long patients had been prescribed the aspirin and statin in between colonoscopies, but could not confirm their adherence. We did not find people who had been prescribed either medication for periods shorter than the interval between procedures, but we assumed that they had been taking the medications daily during that time. Furthermore, we recognize that few observational studies cite nearly a decade of chemoprevention use prior to reduction in CRC mortality [19]. Chung et al. found that 10% of high-risk groups defined as those with AAP or > 2 neoplastic polyps on index colonoscopy developed AAP within 3 years of index colonoscopy [20]. Thus, our primary and secondary outcomes involving AAP provides meaningful findings for a marginalized, relatively high-risk cohort.

Comorbid conditions such as metabolic syndrome requiring therapeutic aspirin and statin chemoprevention for cardiovascular disease are also associated with colorectal neoplasia [21]. This may theoretically limit the treatment effect of chemoprevention in these groups. We acknowledge that some of the AAP identified at subsequent colonoscopy visits were possibly polyps missed on index colonoscopy. Though we attempted to limit this bias by including only colonoscopies with adequate bowel prep ratings, researchers have found through tandem colonoscopy studies that even AAP can be missed in adequately prepped colons [22, 23].

In summary, we provide foundational information regarding the utility of short-term combination of aspirin and statin chemoprevention on AAP incidence among Blacks. There was no difference in AAP development among Blacks taking combined chemoprevention medications over 4 years. We also identified that the presence of AAP on index colonoscopy is a significant risk factor for metachronous incidence of AAP on subsequent colonoscopy among a large Black cohort. The COVID-19 pandemic has negatively impacted the volume of screening colonoscopies [24], so identifying non-invasive methods for CRC risk reduction is pivotal for historically underserved populations. Larger and long-term prospective investigations accounting for comorbidities are needed to evaluate the efficacy of aspirin-statin chemoprophylaxis against CRC in all populations.

## Data Availability

The datasets used and analyzed during the current study are available from the corresponding author on reasonable request.

## Abbreviations

AAP: Advanced adenomatous polyp
APC: Adenomatous polyposis coli
BMI: Body mass index
CRC: Colorectal cancer
EMR: Electronic medical record
HGD: High-grade dysplasia
OR: Odds ratio
